# Acute Effect of High-Intensity Interval Cycling on Carotid Arterial Stiffness and Hemodynamics

**DOI:** 10.1155/2019/6260286

**Published:** 2019-11-20

**Authors:** Wenxue Yuan, Haibin Liu, Zhilin Luan, Zhinan Zhao, Bingyi Shen

**Affiliations:** ^1^Department of Physical Education, Dalian University of Technology, Dalian 116024, China; ^2^Advanced Institute for Medical Sciences, Dalian Medical University, Dalian 116044, China

## Abstract

**Background:**

Cardiovascular disease (CVD) contributes to be one of the leading causes of death in the population worldwide. Carotid arterial stiffness and local hemodynamics are associated with the occurrence and development of CVD. Therefore, understanding the alterations of human carotid arterial stiffness and hemodynamics is of great clinical value in the prevention and treatment of CVD.

**Objective:**

In this study, we aimed to investigate the acute effect of high-intensity interval cycling (HIIC) on carotid arterial stiffness and hemodynamics in sedentary.

**Methods:**

Thirty volunteered healthy sedentary males were enrolled in this study. HIIC intervention (3 sets, 20 s per set) was performed individually. A color Doppler ultrasound was applied to detect the images of the arterial inner diameters and center-line velocity waveforms at the right common carotid artery at different time points (at rest, 3 min, 15 min, and 30 min) after HIIC. Synchronously, electronic manometer was used to measure the systolic and diastolic pressures at the left brachial artery.

**Results:**

Arterial stiffness increased and arterial diameter decreased significantly after acute HIIC. The variation in stiffness persisted for 30 min, at least 15 min longer than the change in diameter. At 3 min after exercise, maximum and mean wall shear stresses (WSS) increased and minimum WSS was also higher than the resting value. At 30 min after exercise, WSS returned to the baseline, but oscillating shear index was still higher than the resting value.

**Conclusions:**

In summary, arterial stiffness and hemodynamics changed significantly not only at 3 min but also at 30 min after acute HIIC.

## 1. Introduction

Cardiovascular disease (CVD) is a significant and ever-growing problem globally, accounting for 31% of mortality [1] higher than malignant diseases and other pathologies. The World Health Organization (WHO) estimates that over 75% of premature CVD is preventable, and protective factors can contribute to reduce the growing burden on both affected individuals and health care systems [2]. Carotid arterial stiffness is closely related to the occurrence and development of atherosclerosis and refers to as the surrogate markers for CVD [3]. Local hemodynamic parameters play a crucial role in regulating the carotid arterial elasticity (stiffness) [4]; therefore, illustrating the parameter alterations of carotid arterial function and hemodynamics may have an advantage in the prediction of CVD and provide valuable clinical guidance to prevent and treat CVD.

Long-term regular aerobic exercise can significantly reduce arterial stiffness and improve arterial structure [5, 6]. In contrast, lack of exercise has been recognized as an important account for increasing morbidity and mortality of CVD [7]. However, in modern society, many young people take a sedentary lifestyle and rarely achieve the minimum amount of exercise recommended by relevant research and administrative organizations. Among all the obstacles, lack of time is the most serious one for the sedentary people to participate in long-term aerobic training. In recent years, a kind of high-intensity interval training has gradually come into public sight. High-intensity interval training generally refers to a form of exercise in which several groups of full-strength, fast, and explosive exercises were performed in a short time [8]. Regular high-intensity interval training can notably improve muscle content and strength, cardiopulmonary endurance, and metabolism [9–13]. Given these characteristics, high-intensity interval training has the potential to be the best choice for sedentary people to meet their exercise needs. However, to date, little attention has been paid to the acute effect of high-intensity interval cycling (HIIC) on arterial structure and function in general population, especially in sedentary population [9, 14, 15].

Carotid artery is one of the main blood supply organs for cerebral circulation system, and its local hemodynamic changes may affect the health condition of intracerebral vascular system [16]. It is well acknowledged that physical exercise can give rise to whole-body or local hemodynamic changes by accelerating blood flow [3]. Endothelial cells covering arteries respond to hemodynamic changes via corresponding receptor sensations and induce the alteration of endothelial cell morphology, function, and gene expression [17]. In recent years, researchers focused on the effects of acute exercise intervention on the function and hemodynamic characteristics of carotid artery. Exercise modes (time, frequency, and intensity) affect the elasticity and hemodynamics of arteries in varying degrees [18–20]. Additionally, Babcock et al. studied the effects of cycling exercise with fixed load on the elastic modulus of carotid artery and local hemodynamics [15]. However, no studies on the effects of acute HIIC on the structure, function, and hemodynamic alterations of carotid artery have been reported in detail.

In the present study, we aimed to investigate the acute effects of HIIC on carotid arterial stiffness and hemodynamic parameters in sedentary population. This study will provide some suggestions for research on the effects of HIIC on arteries and its hemodynamic response and provide advice on the formulation of high-intensity interval exercise program.

## 2. Methods

### 2.1. Subjects

A total of 32 young male adults were recruited in the study (age, 25 ± 3 years; stature, 1.75 ± 0.06 m; mass, 69 ± 5 kg). During cycling intervention, 2 individuals withdrew, due to lack of interest in the study. The entry criteria included sedentary individuals not involved in any regular planned exercise program (exercise less than 2 times a week and less than 15 min each time) in the last 6 months. None of the subjects has been diagnosed with cardiovascular disease, diabetes, respiratory problems, or any related metabolic diseases. Ethical approval was obtained from the Ethics Committee of Dalian University of Technology, and the study was carried out in accordance with the Declaration of Helsinki (1964). Informed consent was acquired from the subjects prior to the onset of HIIC.

### 2.2. Study Design

**Figure 1:**
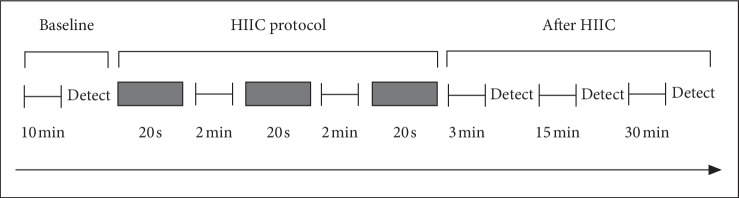
High-intensity interval cycling protocol.

HIIC protocol was applied in the current study. The schematic diagram is shown in Figure 1. Briefly, the naked weight for each subject was firstly measured by electronic scale. After the subjects lie in a supine position for ≥10 min, the hemodynamic parameters of the common carotid artery at rest were evaluated. Then, the subjects completed the warm-up exercise (high-speed tread, 3–5 s each time for 5 min) on cycle ergometer (Powermax-VIII, Combi Wellness, Japan) in order to get familiar with the treading speed requirements for the HIIC. After warm-up exercise, the subjects were asked to take 20-second fast, full-strength, and explosive exercise for three times with an interval of 2 min. The impedance load was set at 7.5% of the body weight for specific subject. At last, the subjects restored to resting state, and the common carotid artery center-line velocity and arterial diameter waveform were measured at 3, 15, and 30 min after exercise, respectively.

### 2.3. Hemodynamic Parameters Measurement

Diameter and center-line velocity waveform of the right common carotid artery were collected by Doppler ultrasound (ProSound Alpha 7, Aloka). Images were acquired 5–10 mm below the carotid bulb. Blood pressure (systolic blood pressure *p*_s_mea_ and diastolic blood pressure *p*_d_mea_) of left brachial artery was collected simultaneously.

### 2.4. Hemodynamic Calculation

#### 2.4.1. Carotid Blood Pressure

In the present study, the mean values of the carotid arterial pressure *p*_m_ and diastolic blood pressure *p*_d_ were considered approximately equal to the mean brachial arterial pressure *p*_m_mea_ and diastolic blood pressure *p*_d_mea_ of left brachial artery [16, 17]. The mean carotid arterial pressure was calculated using the following approximate equation:

(1) $$\begin{array}{c} p_{\text{m}}=p_{\text{m\_mea}}=p_{\text{d\_mea}}+\frac{1}{3}\left(p_{\text{s\_mea}}-p_{\text{d\_mea}}\right). \end{array}$$

Therefore, the carotid artery blood pressure waveform was calibrated using the brachial mean arterial *p*_m_ and diastolic pressure *p*_d_ (=*p*_d_mea_). The maximal value of the carotid artery waveform was calculated and assumed to be the systolic pressure *p*_s_.

#### 2.4.2. Arterial Stiffness

Arterial stiffness refers to the hardenability of arterial blood vessels and was represented by *β*-Stiffness Index (*β*) and calculated by the following equation [21]:

(2) $$\begin{array}{c} \beta =\frac{\ln\left(p_{\text{s}}/p_{\text{d}}\right)}{R_{\text{s}}-R_{\text{d}}}\cdot R_{\text{d}}, \end{array}$$

where *R*_s_ and *R*_d_ are the maximal and minimal carotid diameters.

#### 2.4.3. Flow Rate

Arterial blood flow rate represents the general blood supply of the common carotid artery to the cerebrovascular system in a cardiac cycle and was calculated by the following equation [14]:

(3) $$\begin{array}{c} Q=2\pi {\bar{R}}^{2}\int_{0}^{1}y\cdot u\left(y,t\right)\text{d}y, \end{array}$$

where *R* is the average of the radius of the common carotid artery over time during a cardiac cycle. *y* = *r*/*R*, in which *r* is the radial coordinate.

#### 2.4.4. Peripheral Resistance

The peripheral resistance of common carotid artery reflects the patency of capillaries in the brain and microcirculation in the intracranial vascular bed and was calculated by the following equation [22]:

(4) $$\begin{array}{c} \text{Rq}=\frac{p_{\text{mean}}}{Q_{\text{mean}}}. \end{array}$$

#### 2.4.5. Pulsatility Index (PI)

The pulsatility index is used to express the relationship between blood flow pulsation and arterial pulsation,and was calculated by the following equation:

(5) $$\begin{array}{c} \text{PI}=\frac{V_{\text{max}}-V_{\text{min}}}{V_{\text{mean}}}, \end{array}$$

where *V* is the blood center-line velocity.

#### 2.4.6. Wall Shear Stress (WSS)

The wall shear stress is the frictional force of blood flow acting upon the vascular endothelium and was calculated by the following equation:

(6) $$\begin{array}{c} \tau_{\text{w}}=\frac{\eta }{R}\overset{+\infty }{\underset{n=-\infty }{\sum }}\frac{\alpha_{n}j^{\left(3/2\right)}J_{1}\left(\alpha_{n}j^{\left(3/2\right)}\right)}{J_{0}\left(\alpha_{n}j^{\left(3/2\right)}\right)-1}u\left(0,\omega_{n}\right)e^{j\omega_{n}t}, \end{array}$$

where *α* is harmonic wave, and *η* is blood viscosity. *u*(0, *ω*_*n*_) is the *n* harmonic component of the measured center-line velocities *u*(0, *t*). $j=\sqrt{-1}$. *J*_0_ represents the first kind zero-order Bessel function and *J*_1_ is the first-order Bessel function of the first kind.

#### 2.4.7. Oscillatory Shear Index (OSI)

The oscillatory shear index represents the ratio of retrograde wall shear stress to total wall shear stress in a cardiac cycle. It refers to the oscillation degree of the wall shear stress in both the anterograde and retrograde directions and was calculated by the following equation:

(7) $$\begin{array}{c} \text{OSI}=\frac{1}{2}\left(1-\frac{\left|\int_{0}^{T}\tau_{\text{w}}\text{d}t\right|}{\int_{0}^{T}\left|\tau_{\text{w}}\right|\text{d}t}\right). \end{array}$$

### 2.5. Statistical Analysis

Equations (1)–(7) were programmed and calculated by using Matlab (The MathWorks R2009b, Inc.). Due to the limited experimental conditions, the blood viscosity of each subject could not be measured. The blood viscosity (*η*) was set as 0.004 Pa·s, and the blood density (*ρ*) was determined as 1050 kg/m^3^. The signal of wall shear stress and velocity in the equation were expanded by Fourier series. The maximum harmonic number *n* in the equation was set to 20.

GraphPad Prism 5 (GraphPad Software, Inc.) was applied for data statistics and analysis. All data were presented as the mean ± SD. The repeated ANOVA was used to assess the differences between resting state and 3, 15, and 30 min. When significant differences were detected, Tukey's test was used for post hoc comparisons. The significance level was set at *P*=0.05.

## 3. Results

During cycling intervention of this study, 32 subjects were recruited for the study and 2 people withdrew. As a result, 30 people were statistically analyzed.

### 3.1. Arterial Stiffness and Diameter Responses to HIIC at Different Time Points

**Figure 2:**
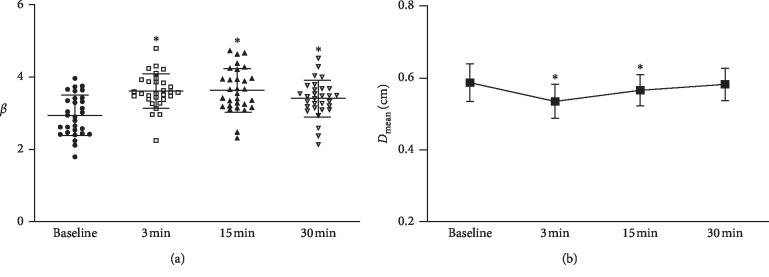
(a) Arterial stiffness (*β*). (b) Mean arterial diameter (*D*_mean_).

Figure 2(a) shows that the arterial stiffness indexes (*β*) at 3 min (3.62 ± 0.48), 15 min (3.64 ± 0.60), and 30 min (3.41 ± 0.50) significantly increased after HIIC compared to *β* at rest (2.95 ± 0.56).  Figure 2(b) indicates that the arterial diameters (*D*_mean_) at 3 min and 15 min significantly decreased after HIIC compared to the arterial diameter at rest.

### 3.2. Carotid Blood Flow Responses to HIIC at Different Time Points

Table 1 lists the changes of carotid blood flow at rest and at different time points after HIIC. Compared with at-rest state, heart rate (HR) increased significantly at 3 min, 15 min, and 30 min after HIIC. Maximum and mean center-line velocities (*V*_max_ and *V*_mean_) at 3 min and 15 min after HIIC were remarkably higher than those at rest. The minimum center-line velocity (*V*_min_) was significantly lower at 3 min than that at rest and showed a negative value. Compared with at-rest state, the maximum blood flow rate (*Q*_max_) was significantly higher, while, the minimum blood flow rate (*Q*_min_) was significantly lower showing a negative value. No significant difference of *Q*_mean_ was shown between any specific time point and at-rest state.

### 3.3. Hemodynamic Responses to HIIC at Different Time Points

**Figure 3:**
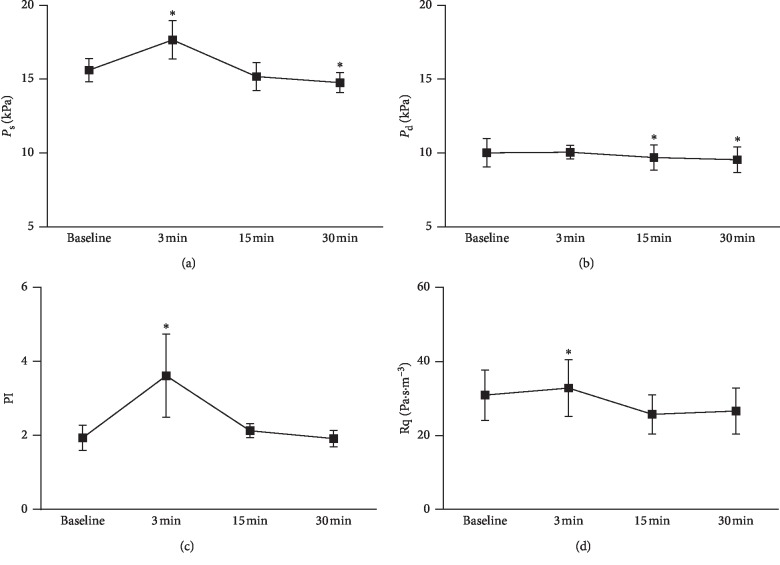
(a) Systolic pressure (*P*_s_); (b) diastolic pressure (*P*_d_); (c) pulsatility index (PI); (d) peripheral resistance (Rq).

As indicated in Figures 3(a) and 3(b), after HIIC, systolic pressure (*P*_s_) significantly increased at 3 min, whereas it decreased at 30 min, compared to that at rest. Diastolic pressure (*P*_d_) significantly decreased at 15 min and 30 min compared to that at rest. Figures 3(c) and 3(d) show that pulsatility index (PI) and peripheral resistance (Rq) were significantly higher at 3 min after HIIC than those at rest.

**Figure 4:**
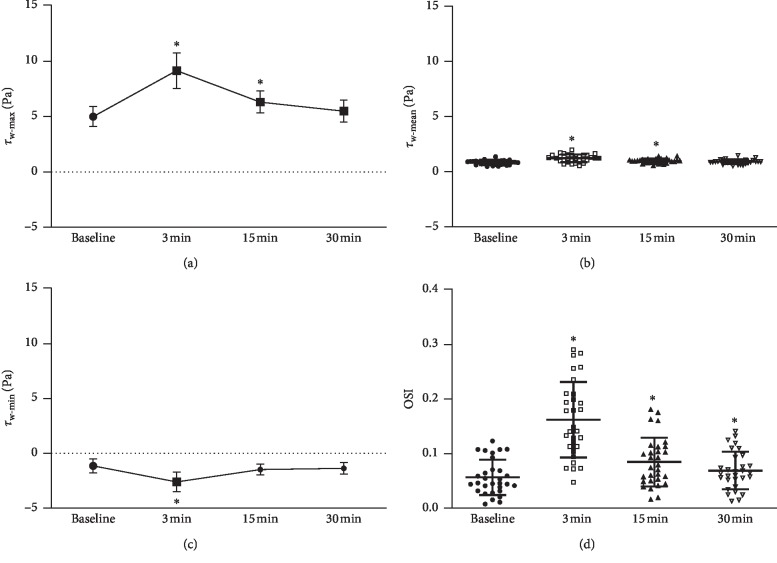
(a) Maximum wall shear stress (*τ*_w_max_); (b) mean wall shear stress (*τ*_w_mean_); (c) minimum wall shear stress (*τ*_w_min_); (d) oscillatory shear index (OSI).

As indicated in Figures 4(a) and 4(c), after HIIC, maximum wall shear stress (WSS) (*τ*_w_max_) significantly increased at 3 min and 15 min, compared to that at rest. Minimum WSS (*τ*_w_min_) at 3 min after HIIC was significantly lower than at rest.  Figure 4(b) indicates that mean WSS (*τ*_w_mean_) at 3 min (1.23 ± 0.35 Pa) and that at 15 min (0.97 ± 0.20 Pa) were both significantly higher than at rest (0.84 ± 0.20 Pa).  Figure 4(d) shows that oscillatory shear index (OSI) at 3 min (0.16 ± 0.07), 15 min (0.08 ± 0.04), and 30 min (0.07 ± 0.03) was significantly higher than that at rest (0.05 ± 0.03).

## 4. Discussion

The aim of the present study was to evaluate the acute effects of HIIC on carotid arterial stiffness and hemodynamic parameters in sedentary people. Through the analysis and calculation of our experimental data, we found that acute HIIC significantly altered the structure and function of common carotid artery. Our results showed increased arterial stiffness and decreased arterial diameter after HIIC, which are consistent with the results reported by Babcock et al. [15]. By evaluating the collected experimental data at 3 min, 15 min, and 30 min after HIIC, we found that the duration of arterial stiffness increase was 30 min after 20-second fast, full-strength, and explosive exercise for three times with an interval of 2 min. We also found that the reduction in diameter lasted 15 min. The transient increase of arterial stiffness induced by acute HIIC may be caused by stress contraction of smooth muscle cells in the middle layer of arteries, which belongs to stress-induced changes in arterial function. In addition, we also discovered that HIIC increased peripheral resistance and pulsatility index of the common carotid artery. Therefore, we can further conclude that acute HIIC may significantly change the resistance characteristics of intracranial peripheral vascular bed, which may pose a risk to sedentary people.

Our study showed that acute HIIC significantly increased arterial systolic pressure, which was consistent with the research conclusion of Rossow et al. [21]. Considering hemodynamic load, acute HIIC is similar to acute impedance exercise. Increased central arterial stiffness and local hemodynamic changes are observed in both conditions. Increased blood pressure induced by acute exercise may alter the elastic structure of blood vessels, leading to vascular sclerosis. It has been reported that upregulated arterial stiffness and endothelial dysfunction are highly correlated with higher systolic pressure responses induced by exercise [23, 24]. Additionally, we further found that systolic and diastolic pressures of the subjects at 30 min after HIIC were lower than those at rest. Previous research has shown that systolic and diastolic pressures decreased at 30 min after moderate-intensity exercise intervention with no significant difference observed [19]. Considering that the similar previous studies only focused on the immediate hemodynamic responses after intensive exercise, hemodynamic parameters were evaluated at 15 min and 30 min after HIIC in the present study [15, 21]. Therefore, we observed the changes of blood pressure at 30 min after HIIC and expanded the study comprehensiveness. Based on the reduction in blood pressure at 30 min after exercise, we can speculate that HIIC has a positive effect in sedentary people on temporarily improving blood pressure.

Significant increases of heart rate and maximum and mean center-line velocities after acute HIIC indicate that HIIC accelerates the flow velocity of common carotid artery. These kinematic characteristics of blood flow are basically consistent with the results of the previous studies on other forms of exercise [15, 25, 26]. Mean blood flow rate is an important index reflecting the state of blood supply in cerebrovascular bed. Regular brain blood supply reduces intake of nonoxidized sugars and increases oxygen supply of the brain. However, our data in this study showed that the increase of maximum and mean center-line velocities has no significant effect on the mean blood flow rate, indicating that total brain blood supply has not increased with HIIC. Furthermore, from the perspective of minimum center-line velocity and minimum flow rate, a “retrograde flow phenomenon” in blood flow of common carotid artery appeared after acute HIIC. This phenomenon may be related to the narrowed arteries and increased peripheral resistance and arterial stiffness. Increased resistance to blood may be the cause of retrograde blood flow. Combined with changes in blood flow rate and direction of blood flow, we can assume that HIIC in sedentary people does not improve blood supply to the brain and may even have negative effects.

Vascular endothelium is a thin layer of endothelial cells covering the inner wall of the artery, which directly contacts with the blood flow. After acute HIIC, the anterograde (positive value) WSS and retrograde (negative value) WSS of common carotid artery increased significantly, indicating that the direction of WSS produced by blood flow continuously changes in arterial vessels. The increase of mean WSS indicates that the amplitude of the change of the anterograde WSS is higher than that of the retrograde WSS, which is consistent with the increase of the oscillatory shear index. It has been acknowledged that WSS plays a crucial role in the process of regulating the structure and function of arteries during exercise [27, 28]. However, the underlying mechanism of the alterations of WSS induced by exercise and its hemodynamics requires further exploration on cellular and physiological levels [29].

High oscillating shear theory established by Ku et al. considered that the high oscillatory shear index may contribute to the atherosclerosis formation [30], which has been questioned by other researches including our current study [31, 32]. In the previous study, our purpose was to compare the acute effects of a cycling intervention on carotid arterial hemodynamics between basketball athletes and sedentary controls [32]. We found that the oscillatory shear index was significantly higher in basketball athletes than that in sedentary people both at rest and after the intervention, and basketball athletes have less stiffness. In order to allow sufficient time to collect the data after each bout of exercise, we chose a longer time interval than that in this study. Comparing the results of the two studies, we can speculate that the longer time interval will attenuate the accumulated effect of multiple and single sets of exercise, with ignoring the effect of exercise intensity. However, the effect of the large-scale wall shear stress oscillation mode on the structure and function of common carotid artery for individuals engaged in long-term and high-intensity interval cycling requires further intensive study to clarify. In this study, our data showed that heart rate and oscillatory shear index at 30 min after HIIC significantly increased compared to the baseline at rest. Therefore, whether the high oscillating wall shear stress induced by HIIC will reduce or increase arterial stiffness during long-term exercise still requires further study.

## 5. Conclusions

In this study, arterial stiffness increased and arterial diameter decreased at 3 min after acute HIIC significantly. Considering these two indicators, we do not recommend that sedentary people start their exercise program with HIIC. In addition, the heart rate and center-line velocity were upregulated with no significant differences in blood supply to brain. Systolic and diastolic blood pressures were downregulated significantly at 30 min after cycling, whereas oscillatory amplitude of blood flow-induced wall shear stress remains higher at 30 min after cycling than that in at-rest state. Acute changes in blood pressure and oscillatory shear index may be beneficial to health, but more studies in chronic effects of HIIC are needed to reveal whether this acute effect could be good for cardiovascular health. In summary, arterial stiffness and hemodynamics changed significantly not only at 3 min but also at 30 min after acute HIIC.

## Figures and Tables

**Table 1:** Variables for carotid blood flow at baseline and after HIIC.

Variables	At-rest state	After HIIC		
		3 min	15 min	30 min
HR (bpm)	66 ± 9	102 ± 10^*∗*^	85 ± 12^*∗*^	77 ± 10^*∗*^
*V*_max_ (m/s)	0.61 ± 0.14	0.99 ± 0.19^*∗*^	0.74 ± 0.13^*∗*^	0.67 ± 0.15
*V*_mean_ (m/s)	0.24 ± 0.05	0.29 ± 0.06^*∗*^	0.29 ± 0.05^*∗*^	0.27 ± 0.05
*V*_min_ (m/s)	0.14 ± 0.03	0.06 ± 0.6^*∗*^	0.12 ± 0.03	0.14 ± 0.02
*Q*_max_ (ml/s)	11.61 ± 3.94	16.49 ± 4.46^*∗*^	13.12 ± 2.71	12.08 ± 3.63
*Q*_mean_ (ml/s)	3.25 ± 0.76	3.20 ± 0.77	3.64 ± 0.69	3.54 ± 0.94
*Q*_min_ (ml/s)	0.72 ± 0.22	−2.67 ± 0.41^*∗*^	0.23 ± 0.31^*∗*^	0.31 ± 0.23

HR, heart rate (bpm); *V*_max_, maximum center-line velocity (m/s); *V*_mean_, mean center-line velocity (m/s); *V*_min_, minimum center-line velocity (m/s); *Q*_max_, maximum blood flow rate (ml/s); *Q*_mean_, mean blood flow rate (ml/s); *Q*_min_, minimum blood flow rate (ml/s).

## Data Availability

All data generated or analyzed during this study are included in this published article.
